# Association of Self-Reported Weight Change and Quality of Life, and Exercise and Weight Management Behaviors Among Adults with Type 2 Diabetes Mellitus: The SHIELD Study

**DOI:** 10.1155/2012/892564

**Published:** 2012-05-08

**Authors:** Susan Grandy, Kathleen M. Fox, Debbra D. Bazata

**Affiliations:** ^1^AstraZeneca LP, Wilmington, DE 19850-5437, USA; ^2^Strategic Healthcare Solutions, LLC, Monkton, MD 21111-0543, USA; ^3^St. Luke's Primary Care South, Overland Park, KS 66213, USA

## Abstract

*Purpose*. This study examined the association between self-reported weight change and quality of life, and exercise and weight management behaviors among individuals with type 2 diabetes mellitus (T2DM). *Methods*. In the US SHIELD study, respondents reported whether they had lost or gained weight compared with 1 year earlier and completed the SHIELD-WQ-9 quality of life questionnaire as well as provided information on their exercise and weight management behaviors in the past 12 months. *Results*. Sixteen percent of the respondents reported gaining weight (*n* = 460), and 30% reported losing weight (*n* = 895). More respondents who reported losing weight exercised regularly, limited calorie and fat intake, and increased fiber, fruit, and vegetable intake compared with respondents who reported gaining weight (*P* < 0.01). For all nine aspects of daily life, a significantly greater proportion of respondents who reported losing weight reported improved well-being (12%–44%) compared with respondents who reported gaining weight (*P* < 0.0001). *Conclusions*. Self-reported weight loss was associated with improved well-being, better exercise, and weight management behaviors among individuals with T2DM.

## 1. Introduction

During the past decade, there has been a dramatic increase in diabetes and obesity in the United States [1]. In the United States, there are 23.5 million adults aged 20 years or older with diabetes [2]. Worldwide, 366 million people have diabetes in 2011, with the number of people with type 2 diabetes mellitus (T2DM) increasing in every country [3]. The increasing prevalence of T2DM is directly related to an increasing rise in the prevalence of obesity and physical inactivity, with an estimated 97 million US adults being overweight or obese [4, 5]. National surveys report that 66% of Americans are overweight (body mass index (BMI) = 25.0–29.9 kg/m^2^), and 32% are classified as obese (BMI ≥30 kg/m^2^) [1, 6, 7]. More than 1.1 billion adults worldwide are overweight, and 312 million are obese [8]. Approximately 40%–50% of patients with diabetes also are obese [9].

Weight loss has been associated with reduced insulin resistance and improvements in glycemia [9]. Weight management is a key self-management treatment for individuals with T2DM. The American Diabetes Association (ADA) Standards of Medical Care in Diabetes [10] and International Diabetes Federation [3] recommend weight loss for all overweight or obese individuals who have T2DM to assist in achieving and maintaining glycemic control. Despite the recommendations, information on health outcomes associated with weight change is often limited for individuals with T2DM. Cross-sectional studies among the general population have shown a clear inverse relationship with baseline weight or BMI and health-related quality of life (HRQOL) [11–13]. Studies examining the change in weight over time and HRQOL found that weight gain led to a reduction in HRQOL [14, 15], and in general, weight loss (5%–10%) was associated with significant improvement in HRQOL, typically in physical functioning and bodily pain [14, 16–18]. However, only one study examined this association among individuals with T2DM [19], where the results indicated that patients with diabetes who lost >5% of their weight during the clinical trial had improved their HRQOL scores.

For observational studies, methodological difficulties in assessing the association between weight change and HRQOL include the selection of the HRQOL instrument (condition specific or generic) and the timing of the HRQOL assessment in relation to the weight change. If the HRQOL instrument does not refer to weight or weight change, respondents may answer based on other health conditions that affect their HRQOL, and the association with weight change may be confounded. If a generic health instrument is used and the weight change occurred in the past before the recall period of the instrument, respondents may not consider their weight or change in weight when answering the survey. The present study examined the association between self-reported weight change and well-being utilizing the SHIELD-WQ-9, a new questionnaire developed to link HRQOL assessment to change in weight.

## 2. Methods

The present investigation is a cross-sectional analysis of data from the Study to Help Improve Early evaluation and management of risk factors Leading to Diabetes. (SHIELD) assessing the association between weight change and HRQOL. SHIELD is a 5-year, survey-based study conducted to better understand patterns of health status, health behavior, and knowledge and attitudes of people living with diabetes and those with varying levels of cardiometabolic risk.

### 2.1. SHIELD Survey

SHIELD included an initial screening phase to identify cases of interest in the general population (e.g., diabetes mellitus), a baseline survey to follow up identified cases with a questionnaire about health status, health knowledge and attitudes, current behaviors and treatments, and annual follow-up surveys. A detailed description of the SHIELD methodology has been published previously [20, 21].

In brief, the screening survey was mailed in April 2004 to a stratified random sample of 200,000 US households, representative of the US population for geographic residence, household size and income, and age of head of household [22], identified by the Taylor Nelson Sofres National Family Opinion (TNS NFO) panel (Greenwich, CT). All TNS NFO surveys were voluntary, and no special incentives were provided. A response rate of 64% was obtained for the screening survey. The SHIELD study was approved by the Quorum Review Board.

A comprehensive baseline survey was mailed in September-October 2004 to a representative sample of individuals (*n* = 22, 001) who were identified in the screening survey as having self-reported type 1 diabetes mellitus or type 2 diabetes mellitus, no diabetes, or being at risk for diabetes. Each respondent group was balanced to be representative of that segment of the population for age, gender, geographic region, household size, and income for the US population, and then a random sample from each group was selected and sent the baseline survey. A response rate of 72% was obtained for the baseline survey. The 2008 annual follow-up survey (response rate of 71%) included the SHIELD-WQ-9 questionnaire on weight change and how the weight change affected nine aspects of daily life as well as the SF-12 quality-of-life questionnaire. Responses from the 2008 survey were analyzed and reported in this study.

### 2.2. Study Measures

Respondents were classified as having T2DM based upon their self-report of having been told by a doctor, nurse, or other healthcare professional that they had T2DM. Other cardiovascular comorbid conditions were identified in a similar manner, with respondents having been told by a healthcare professional that they had cholesterol problems, circulation problems of any kind, heart disease or heart attack, hypertension, or narrow or blocked arteries.

For weight change, respondents were asked to compare their current weight with their weight 1 year ago and to indicate if they had gained weight, lost weight, stayed the same, lost weight then gained it back, or gained weight but lost it again. Utilizing the SHIELD-WQ-9, respondents were then asked the following: “thinking about your weight change or lack of weight change over the past year, how did this change (or lack of change) affect you in the following areas? In the past year, my change or lack of change in weight had the following effects.” Response categories were “worsen, improved, stayed the same, and not applicable.” The 9 following areas were reported: (1) how I feel physically (physical health), (2) my interactions with family, (3) my work performance, (4) my interactions with coworkers and friends, (5) my social activities, (6) my daily activities, (7) my self-esteem, (8) how I feel emotionally (emotional health), and 9) my overall quality of life. The SHIELD-WQ-9 was developed to assess HRQOL specific to changes in weight using the same domains (e.g., physical health and mental health), similar to other HRQOL instruments like the SF-36 and SF-12.

Exercise behaviors were reported based upon responses to a general exercise question and the International Physical Activity Questionnaire (IPAQ). First, respondents were asked to describe their exercise routine as “currently exercise regularly” versus “currently exercise some” or “currently do not exercise”. Additionally, the type and length of physical activity over the previous 7 days were assessed through the 7-item IPAQ short form [23]. The IPAQ scores are categorized into 3 levels: (1) low or inactive, (2) moderate activity of at least 600 metabolic equivalent (MET) minutes/week (3 or more days of vigorous activity of at least 20 minutes per day or 5 or more days of moderate activity or walking of at least 30 minutes per day), and (3) high activity of a minimum of 1500 MET minutes/week (vigorous activity on at least 3 days or 7 days of any combination of walking and moderate or vigorous activities). Weight management behaviors included responses to “have you tried to lose weight during the past 12 months” (yes/no), “have you done anything to keep from gaining weight during the past 12 months” (yes/no), “how often do you weigh yourself” (times per month), “how many times a week do you usually eat at a fast food restaurant,” “which of these are you currently trying to do” (trying most of the time or trying very hard/every day versus trying some/little of the time or do not try to do), limit calories, eat less fat, eat more fiber, limit amount of meat, eat more fruit, and eat more vegetables. Weight and height was self-reported at the time of the survey. Actual weight change was computed by subtracting weight as reported in the 2007 survey from weight reported in the 2008 survey.

### 2.3. Statistical Analysis

The proportion of respondents reporting weight loss or weight gain was computed for respondents with T2DM. Comparisons between T2DM respondents who reported losing weight and those who reported weight gain were made using chi-square tests. Statistical significance was set *a priori* as *P* < 0.05.

## 3. Results

A total of 3,000 respondents with T2DM responded to the 2008 survey, of which 2,969 respondents answered the self-reported weight change question. Of the 2,969 T2DM respondents, 15.5% reported gaining weight over the past year, 30.1% reported losing weight, 41.1% reported staying the same, and 13.2% reported fluctuating weight. The analysis included those respondents reporting weight gain (*n* = 460) or weight loss (*n* = 895) and excluded those not reporting weight change or consistent weight change.

Respondents who reported weight loss were similar to respondents who reported weight gain with regard to gender (61% versus 64% women), race (75% versus 71% white), household income, and education (*P* > 0.10) (Table 1). Additionally, cardiovascular comorbid conditions (e.g., cholesterol problems and heart disease) were similar between respondents who reported losing weight and those who reported gaining weight. However, those who reported weight loss were older (on average 3 years) and weighed less at the time of the survey than respondents who reported weight gain (*P* < 0.05). A greater proportion of respondents who reported weight gain were obese (BMI ≥ 30 kg/m^2^), compared with those who reported weight loss (*P* < 0.001). Using the reported weight in 2007 and 2008 to calculate weight change rather than self-reported weight change, 75% of respondents who reported losing weight actually did lose weight, an average of 9 pounds (Table 1). Similarly, 70% of respondents who reported weight gain actually did gain weight, an average of 7 pounds. The correlation coefficient for the comparison of self-reported weight change with actual weight change was 0.56 (*P* < 0.001).

### 3.1. Exercise and Weight Management Behaviors

A greater proportion of respondents who reported losing weight (30%) also reported exercising regularly compared with respondents who reported gaining weight (18%, *P* < 0.001) (Table 2). Likewise, more respondents who reported weight loss were highly active and fewer were inactive based on IPAQ scores, compared with respondents who reported weight gain (*P* < 0.0001). Positive weight management behaviors (tried to lose weight, keep from gaining weight) were more evident among respondents who reported weight loss compared with respondents who reported weight gain. A greater proportion of respondents reporting weight loss reported doing things to keep from gaining weight (78%) compared with respondents who reported weight gain (70%, *P* = 0.001). Respondents who reported weight loss indicated they weighed themselves more frequently than respondents who reported weight gain; 35% of respondents with reported weight loss weigh themselves 6 or more times per month versus 24% of respondents with reported weight gain (*P* = 0.001). Eating at fast food restaurants was more frequent among respondents who reported weight gain (20% ate out 3 or more times per week) than respondents who reported weight loss (11%, *P* = 0.006). A greater proportion of respondents who reported weight loss indicated they had improved their diet by limiting calories, eating less fat, eating more fiber, limiting the amount of meat, and eating more fruits and vegetables, compared with respondents who reported weight gain (*P* < 0.01 for each). 

### 3.2. Well-Being with SHIELD-WQ-9

A significantly greater proportion of respondents who reported losing weight reported improved well-being for all 9 aspects of daily life compared with respondents who reported gaining weight (Table 3). Forty-four percent of respondents who reported losing weight reported improved physical health, compared with 3% of respondents who reported gaining weight (*P* < 0.001). Similarly, 32%–39% of respondents who reported losing weight reported improved emotional health, self-esteem, and overall quality of life, compared with 5% of respondents who reported gaining weight (*P* < 0.001). Twenty-six percent of respondents who reported losing weight reported improvement in daily activities, compared with 4% of respondents who reported gaining weight (*P* < 0.001). Likewise, 12%–18% of respondents who reported losing weight reported improvement in interactions with family, coworkers, and friends, work performance, and social activities, compared with 3%–5% of respondents who reported gaining weight (*P* < 0.001). 

For respondents reporting weight loss, the greatest proportions indicating improvement were for physical health (44%), self-esteem (39%), and overall quality of life (34%). Only 5% or less of the respondents reporting weight gain indicated improvement in any of the 9 aspects of daily life. 

## 4. Discussion 

Self-reported weight loss, compared with self-reported weight gain, was associated with improved well-being among individuals with T2DM. A significantly larger percentage of respondents reporting weight loss indicated improvement in all 9 aspects of daily life than respondents reporting weight gain. Additionally, respondents reporting weight loss had significantly better exercise and weight management behaviors. Respondents reporting weight loss exercised and were more physically active than respondents who reported weight gain. Respondents reporting weight loss indicated they managed their weight and diet better than respondents reporting weight gain by monitoring their weight more frequently, trying to keep from gaining weight, eating less frequently at fast food restaurants, and eating healthier. 

The evaluation of HRQOL in this study was performed using a newly developed, weight-related questionnaire (SHIELD-WQ-9). The SHIELD-WQ-9 was specifically developed to elicit HRQOL based on changes in weight, which may represent a truer impact of weight change on HRQOL than other general HRQOL instruments like the SF-36. Generic HRQOL instruments are not specifically targeted to weight change but overall quality of life, which most likely was influenced by more significant health-related issues than weight change, such as diabetes and/or cardiovascular disease, since 73%–77% of respondents had cholesterol problems and hypertension and 24%–26% had heart disease or heart attack. Further research is needed to validate the instrument in assessing weight-related HRQOL among individuals with diabetes as well as other health conditions. 

This study adds to the evidence base for an association between weight loss and better HRQOL among individuals with T2DM. Very few studies have examined this relationship for adults with T2DM [19], and no study has been done on a large population-based sample of T2DM. The present study found a similar association of weight loss and improvement in HRQOL as other studies among the general population [14, 16–18]. With this evidence that weight loss is associated with improved HRQOL among individuals with T2DM, it is important to determine if the improved HRQOL along with the weight loss will assist T2DM patients to better manage their diabetes (possibly improve medication adherence and glucose monitoring) and control their glucose levels. Information about glycemic control (HbA1c levels) was not collected in the SHIELD survey; thus, this study was not able to investigate whether respondents who reported weight loss had better glycemic control. Future research is needed to further evaluate whether weight loss has a positive impact on diabetes self-management leading to better health outcomes. 

Self-reported weight loss was also associated with better exercise and weight management behaviors. Respondents who reported weight loss also reported exercising more and eating better than respondents who reported weight gain. These lifestyle behaviors need to be encouraged and supported by family and the healthcare system so that adults with T2DM can successfully manage their weight and reduce the risk of obesity, which is at epidemic rates among individuals with T2DM. It is important to note that only 30% of respondents who reported weight loss indicated they exercised regularly and only 13% were highly active based on IPAQ scores. These figures indicate a large gap in lifestyle management even among those who reported weight loss. There is a large portion of respondents among those reporting weight loss who are not physically active or do not exercise regularly (60%–70%). The gap in weight management is not as large, but only 51%–62% of respondents reporting weight loss reported limiting calories, eating less fat, and eating more fiber, leaving 38%–49% of respondents not adopting healthy lifestyle behaviors. These gaps in positive exercise and weight management behavior need to be narrowed so that greater improvements in HRQOL and other positive health outcomes can be experienced by more adults with T2DM. 

There are limitations to the study that should be considered. The determination of T2DM and weight was made based upon self-report rather than clinical or laboratory measures. However, actual weight and weight change were captured, and the majority of respondents (75%) did report their weight change accurately. The SHIELD-WQ-9 continues to be administered in different T2DM treatment settings to test its validity and reliability. Household panels, like the SHIELD study, tend to underrepresent the very wealthy and very poor segments of the population and do not include military or institutionalized individuals. However, these limitations are true for most random sampling and clinically based methodologies. Self-selection bias may be present because respondents were those who could read and comprehend the survey.

## 5. Conclusions 

Self-reported weight loss, compared with self-reported weight gain, was associated with improved well-being and positive exercise and weight management behaviors among individuals with type 2 diabetes. 

## Figures and Tables

**Table 1:** Characteristics of SHIELD respondents with type 2 diabetes who reported gaining or losing weight in the past year.

Characteristics	Lost weight (*n* = 895)	Gained weight (*n* = 460)
Age, years, mean (SD)	63.0 (11.8)*	60.0 (10.6)
Women, %	61.1	63.9
White, %	74.6	70.7
Income, % with ≤$35,000/year	34.9	36.1
Education, % with no more than a high school degree	31.7	36.4
Cholesterol problems, %	73.5	76.7
Circulation problems of any kind, %	19.9	23.0
Heart disease/heart attack, %	26.3	23.7
Hypertension, %	73.0	76.7
Narrow or blocked arteries, %	10.3	7.6
Weight, lbs, mean (SD)	209.7 (54.7)*	230.9 (60.8)
Body mass index (BMI) category, %	*	
Normal weight (BMI < 25.0 kg/m^2^)	11.8	3.8
Overweight (BMI = 25.0–29.9 kg/m^2^)	25.6	19.6
Obese (BMI ≥ 30 kg/m^2^)	62.6	76.7
Calculated weight change from 2007 to 2008 in pounds, mean (SD)	−9.2 (17.0)*	7.1 (16.7)
Lost weight from 2007 to 2008, %	75.4*	20.3
Gained weight from 2007 to 2008, %	15.8*	69.5
No change in weight from 2007 to 2008, %	8.8	10.2

*P < 0.05 for comparison of lost weight versus gained weight; 1 pound = 0.4536 kg.

**Table 2:** Exercise and weight management behaviors among respondents with type 2 diabetes who reported gaining or losing weight in the past year.

	Lost weight (*n* = 895)	Gained weight (*n* = 460)
Exercise behavior		
Currently exercise regularly, %	30.0*	17.7
International Physical Activity Questionnaire score	*	
Inactive/low activity	62.7	73.8
Moderately active	24.1	17.3
Highly active	13.2	8.9
Weight management behavior		
Tried to lose weight in past 12 months, %	70.8	66.7
Done things to keep from gaining weight in past 12 months, %	78.2*	69.6
How often do you weigh yourself each month?	*	
0 times per month	15.1	25.4
1 time	18.5	21.7
2–5 times	31.4	28.8
6–15 times	12.8	10.7
16 or more times	22.3	13.4
Number of times per week that you eat at a fast food restaurant, %	*	
0 times per week	42.4	33.7
1	31.7	31.9
2	14.8	14.1
3–15	11.1	20.3
Proportion who try to do the following most of the time or every day, %		
Limit calories	50.7*	36.1
Eat less fat	61.6*	50.7
Eat more fiber	57.1*	47.6
Limit meat	36.9*	30.3
Eat more fruit	63.8*	51.4
Eat more vegetables	68.2*	55.8

*P < 0.01 for comparison of lost weight versus gained weight.

**Table 3:** Proportion of respondents with type 2 diabetes who self-reported (SHIELD-WQ-9) improvement in quality of life over the past year related to their weight change.

Respondents indicating improvement over the past year (%)	Lost weight* (*n* = 895)	Gained weight (*n* = 460)
Physical health	44.0	3.1
Interactions with family	17.6	5.3
Work performance	17.7	2.7
Interactions with coworkers/friends	12.5	2.7
Social activities	16.8	2.9
Daily activities	25.5	3.6
Self-esteem	39.4	4.9
Emotional health	31.9	4.9
Overall quality of life	34.2	4.9

*P < 0.001 for comparison of lost weight group versus gained weight group.
